# Dynamic and specific immune responses against multiple tumor antigens were elicited in patients with hepatocellular carcinoma after cell-based immunotherapy

**DOI:** 10.1186/s12967-017-1165-0

**Published:** 2017-03-22

**Authors:** Yanyan Han, Yeting Wu, Chou Yang, Jing Huang, Yabing Guo, Li Liu, Ping Chen, Dongyun Wu, Junyun Liu, Jin Li, Xiangjun Zhou, Jinlin Hou

**Affiliations:** 1 State Key Laboratory of Organ Failure Research, Guangdong Provincial Key Laboratory of Viral Hepatitis Research, Department of Infectious Diseases, Nanfang Hospital, Southern Medical University, Guangzhou, China; 2 HRYZ Biotech Co., Shenzhen, China

**Keywords:** Tumor associated antigens, Immune responses, Liver cancer, Dendritic cell vaccine, Adoptive cell therapy

## Abstract

**Background:**

Hepatocellular carcinoma (HCC) is one of the most common cancers in China and frequently occurs with chronic hepatitis B virus infection. To investigate whether cell-based cancer immunotherapy induces tumor specific immune responses in patients with HCC and provides clinical benefits, as well as to elucidate the most immunogenic tumor associated antigens (TAAs), multiple antigen stimulating cellular therapy (MASCT) was applied in addition to standard of care.

**Methods:**

Mature dendritic cells (DCs) and activated T cells prepared for MASCT were generated from autologous peripheral blood mononuclear cells (PBMCs). DCs were loaded with a peptide pool of multiple HCC-related tumor antigens, and T cells were stimulated by these DCs.

**Results:**

Thirteen patients with HCC received repeated MASCT after tumor resection during which their immune responses were examined. After three courses of MASCT, the frequency of regulatory T cells in the patients’ PBMCs significantly decreased (*p* < 0.001), while the antigen peptide pool-triggered T cell proliferation (*p* < 0.001) and IFNγ production (*p* = 0.001) were significantly enhanced. The specific T cell responses against each antigen in the pool were detected in 11 patients, but with individualized distinct patterns. The most immunogenic TAAs for HCC are survivin, CCND1, and RGS5. Moreover, the antigen-specific immune responses observed in tumor-free patients’ PBMCs were significantly stronger than that in the patients with recurrence (*p* = 0.037).

**Conclusions:**

Our study demonstrates that MASCT is well-tolerated by patients with HCC and elicits strong and dynamic immune responses specifically against multiple tumor associated antigens, which may correlate with clinical outcomes.

**Electronic supplementary material:**

The online version of this article (doi:10.1186/s12967-017-1165-0) contains supplementary material, which is available to authorized users.

## Background

According to the newly released cancer statistics in China, 2015, hepatocellular carcinoma (HCC) is the fourth most common cancer and the third leading cause of mortality [1]. With the high frequency of chronic hepatitis B virus (HBV) infection, more than 100 million people are facing high risks of developing HCC later in their lives. The therapeutic options for HCC patients with advanced disease are extremely limited. Current conventional therapies such as resection, liver transplantation, and transcatheter arterial chemoembolization (TACE) are utilized to treat patients with early stage cancer, even though HCC is rarely cured and usually relapses quickly. Alternative therapies are urgently needed for HCC patients.

Cell-based cancer immunotherapies including dendritic cell (DC)-based therapeutic cancer vaccines [2] and adoptive cell therapies (ACT), have been considered as effective options to treat cancer patients for decades, particularly for patients with late stage diseases. Clinical responses, including complete tumor recession and long-term disease-free survival, have been observed in patients with metastatic melanoma as well as other types of cancer [3]. These clinical benefits are correlated with, or resulting from, the existence of tumor-specific T cells [4, 5], which are induced in vivo after DC vaccine, or selectively activated and amplified ex vivo and infused during ACT [4, 6]. Recently, immune checkpoint blockades such as anti-CTLA4, anti-PDL1, and anti-PD1 monoclonal antibodies have shown exciting clinical benefits in diverse solid cancers through a molecular mechanism depending on the pre-existing tumor-specific T cells [7]. Therefore, to select the most immunogenic TAAs for HCC and elicit tumor-specific T cell responses in HCC patients by cell-based immunotherapy is an attractive strategy.

In this study, we treated 13 HCC patients with multiple antigen stimulating cellular therapy (MASCT) after tumor resection. During MASCT, mature DCs pulsed with a peptide pool of multiple tumor antigens and autologous T cells stimulated with theses DCs followed by ex vivo proliferation were sequentially injected to patients with HCC to elicit both active and passive immune responses in vivo. The major purpose of this study was to investigate the mechanism and outcomes of using multiple tumor antigens in MASCT and to provide a safe cell-based cancer immunotherapy to prevent HCC recurrence in tumor-free patients.

## Patients and methods

### Patients and sample preparation

Thirteen patients with HCC were enrolled in this study and received MASCT in the center of liver diseases, Nanfang hospital, Southern Medical University, Guangzhou, China. All of them were tumor-free before MASCT. All patients signed informed consents before MASCT was administered. The eligible criteria included: an Eastern Cooperative Oncology Group performance status score of no more than 2, a life expectancy of more than 3 months, no severe cardiovascular disease, no autoimmune disease, and no pregnancy. Patients stopped MASCT and received standard of care when tumor recurred.

PBMCs were collected and frozen in liquid nitrogen at the following time points: (1) at baseline when the first apheresis was performed (week 1); (2) after the second injections of mDCs (week 6); (3) after the first injection of activated T cells (week 10); (4) after the first course of MASCT (week 16); (5) after the second course of MASCT (week 31); (6) 2 months after finishing the third course of MASCT (week 52). PBMCs were thawed to perform the following experiments: regulatory T cell detection, antigen-specific T cell proliferation, antigen-specific IFNγ production, and ELISPOT assay, when the patient had completed or stopped his/her MASCT. This study protocol conformed to the ethical guidelines of the 1975 Declaration of Helsinki as reflected in a priori approval by the ethics committee of Nanfang hospital, Southern Medical University, Guangzhou, China.

### Cell preparation for MASCT

PBMCs from patients with HCC were obtained by density gradient centrifugation using Lymphoprep (NycomedPharma, Oslo, Norway). Adherent monocytes were cultured in AIM-V (Gibco, Carlsbad, CA) with GM-CSF (1000 U/mL) and IL-4 (500 U/mL) to differentiate into immature DCs. These immature DCs were then pulsed by a peptide pool of multiple tumor antigens (1 μg/mL/peptide), followed by culture with a maturing cocktail (IL-6, 1000 U/mL; TNF-α, 1000 U/mL; IL-1β, 10,000 U/mL; PEG2, 1 μg/mL; Poly I:C, 10 μg/mL), to differentiate into antigen-presenting mature DCs. To prepare the activated T cells for infusions, either the frozen non-adherent PBMCs were thawed or the freshly isolated PBMCs were co-cultured with antigen loaded mature DCs for about 4 weeks in the presence of IL-2 (1000 U/mL). The anti-CD3 antibody (50 ng/mL) was added 3 days after co-culturing.

The peptide pool of multiple tumor antigens used in MASCT consisted of 12 TAAs, which were overexpressed in cancerous hepatocytes and two HBV associated antigens (Table 1; Additional file 1: Table S1). The irrelevant peptide was from the envelope of HIV. All peptides were chemically synthesized under GMP conditions.

**Table 1:** Composition and characteristics of the HCC antigen peptides pool

No.	Antigen	Overexpressed in HCC	Clinical trials^a^	References
1	hTERT	+	OC, BC, PC, melanoma, MM	[8, 9]
2	p53	+ (loss of function)	LC, BC, OC, melanoma, PC, HNSCC, CRC, CC	[10, 11]
3	Survivin	+	HCC, AML, ALL, OC, BC, PC, RCC, melanoma, MM, LC, EC, STS	[10–12]
4	NY-ESO-1	+	HCC, SS, MM, melanoma, OC, LC, oesophageal cancer; sarcoma, BC, bladder carcinoma	[8, 13]
5	CEA	+	CRC, LC, BC, GC, LM	[14, 15]
6	CCND1	+	RCC	[16, 17]
7	c-MET	+	RCC, BC	[17, 18]
8	RGS5	+	RCC	[17, 19]
9	MMP7	+	RCC	[17, 20]
10	VEGFR	+	Melanoma, RC, RCC	[21]
11	AFP	+	HCC	[8, 22]
12	GPC3	+	HCC	[23, 24]
13	HBV core antigen	+ (when HBV^+^)	–	[8, 17]
14	HBV DNA polymerase	+ (when HBV^+^)	–	

*hTERT* human telomerase reverse transcriptase, *CEA* carcinoembryonic antigen, *CCND1* cyclin D1, *MET* HGF-hepatocyte growth factor receptor, *RGS5* regulators of G protein signaling 5, *MMP7* matrix metalloproteinase 7, *VEGFR* vascular endothelial growth factor receptor, *AFP* alpha fetoprotein, *GPC3* glypican-3, *OC* ovarian cancer, *BC* breast cancer, *PC* pancreatic cancer, *MM* multiple myeloma, *LC* lung cancer, *HNSCC* head and neck squamous cell carcinoma, *CRC* colorectal cancer, *CC* cervical cancer, *HCC* hepatocellular carcinoma, *AML* acute myeloid leukemia, *ALL* acute lymphoblastic leukemia, *RCC* renal carcinoma, *EC* esophagus cancer, *STS* soft tissue sarcoma, *SS* synovial sarcoma, *GC* gastric cancer, *LM* liver metastases, *RC* renal cancer

^a^Only the clinical trials of cancer immunotherapies such as DC vaccines, ACT and peptides vaccines were indicated

### Immunofluorescence

DCs were cultured in chamber slides (ThermoScientific, Waltham, MA) and pulsed with FITC-labeled peptides. After 2 h, DCs were labeled with DAPI (Molecular Probes, Eugene, OR, USA) and LysoTracker (Molecular Probes, Eugene, OR, USA) to identify the nuclei and lysosomes, respectively. The fluorescence images were recorded using a confocal laser scanning microscope (TCS SP5II, Leica Microsystems GmbH, Wetzlar, Germany).

### Flow cytometric analysis

Antibodies for surface markers staining of DCs and T cells were obtained from BD Biosciences, New York, USA (anti-human CD3-PE, CD3-FITC, CD8-PerCP, CD8-APC, CD56-PE, NKG2D-APC, CD4-FITC, CD4-PerCP, CD107a-FITC, CD25-APC, CD45RO-FITC, CD27-PerCPCY5.5, CD57-APC, CCR7-PE, CD14-APC, CD80-PE, CD83-APC, CD86-FITC, HLA-DR-FITC). Antibodies for intracellular proteins staining were also obtained from BD Biosciences (anti-human IFNγ-APC, TNFα-PECY7, granzyme B-FITC, FoxP3-PE). The intracellular staining was performed by fixing and permeabilizing cells with Cytofix/Cytoperm (BD Biosciences). The experiments were performed by using FACS CantoII (BD Biosciences) flow cytometer, and data were analyzed by using the Flowjo software.

### In vitro cell killing assay

HepG2 and Huh7 cells were cultured in DMEM (Gibco, Carlsbad, CA) supplemented with 10% inactivated fetal bovine serum. The cell killing assay was performed according to the manufacturer’s instructions of the Cytotoxic 96 Assay kit (Promega G1780, Madison, WI). Briefly, HepG2 and Huh7 cells were washed with D-PBS (Gibco, Carlsbad, CA) and co-cultured with the activated T cells, in triplicates, for 4 h before detection. Different effector to target cell ratios (E:T) were performed such as 40:1, 20:1, 10:1, and 5:1. Cytotoxicity is presented as the percentage of maximum LDH release after lysis.

### Proliferation assay and IFNγ production of antigen-specific T cells

PBMCs from patients were plated (1 × 10^6^ cells/well) in AIM-V containing IL-2 (50 U/mL) and stimulated with the peptide pool for 3 days. To determine the percentage of proliferating T cells, Click-iT EdU Alexa Fluor 488 Flow Cytometry Assay (Invitrogen, Carlsbad, CA) was performed according to the manufacturer’s instructions and analyzed by flow cytometry. The IFNγ production of antigen-specific T cells was detected by intracellular staining and analyzed by flow cytometry. PBMCs incubated with irrelevant peptide at the same concentration were used as internal controls. The results are presented as fold changes of antigen peptide pool stimulation compared to irrelevant peptide stimulation.

### ELISPOT

Patients’ PBMCs were first plated (1 × 10^6^ cells/well) in 96-well plate in AIM-V and stimulated with the antigen peptide pool, each antigen peptide (10 μg/mL), or irrelevant peptides for 48 h. These PBMCs were then transferred onto 96-well ELISPOT assay plate (U-CyTech Biosciences, Utrecht, Netherlands) and stimulated again with peptides for another 16 h for IFNγ detection. The ELISPOT assay was performed and analyzed according to the manufacturer’s instructions. The number of spot-forming units was determined by computer-assisted image analysis software (ChampSpot; Saizhi). The responses are represented as spot-forming units per 2 × 10^5^ PBMCs/well.

## Results

### MASCT treatment for patients with HCC

**Fig. 1:**
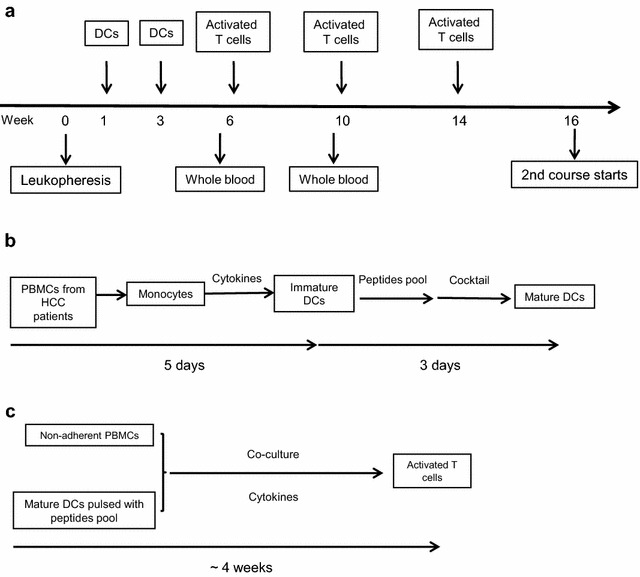
The schematic diagram of one course of MASCT treatment (**a**), the manufacturing process of mature DCs (**b**), and the activated T cells (**c**)

Thirteen patients with HCC, 12 of Barcelona Clinic Liver Cancer (BCLC) stage A and one with BCLC stage C disease, who underwent surgery for tumor resection were recruited. All of them were diagnosed as tumor-free by computed tomography (CT) scan after the surgery. Most of them presented with normal liver function, renal function, tumor serum biomarkers, and HBV DNA copies at baseline before MASCT, except patient No. 8 (Additional file 1: Table S2). Each patient was required to complete three courses of MASCT if no tumor-recurrence was detected. Otherwise, the patient was asked to stop MASCT and received standard of care. Therefore, only ten patients finished two courses of MASCT and only eight patients completed three courses of MASCT. During each course, patients received two subcutaneous injections of mature DCs pulsed with a peptide pool consisting of multiple HCC-related tumor antigen epitopes (Additional file 1: Table S1), and three intravenous injections of activated T cells stimulated by antigen peptides loaded DCs. Each course lasted 14 weeks, and the interval was 2 weeks (Fig. 1a). The manufacturing processes of mature DCs and activated T cells are described in “Patients and methods” and shown in Fig. 1b, c.

The peptide pool of multiple tumor antigens (Table 1) included ten TAAs, which are overexpressed in malignant cells from various cancers, including HCC such as survivin, NY-ESO-1, and carcino-embryonic antigen (CEA); two TAAs, alpha fetoprotein (AFP) and glypican-3 (GPC3), which are specifically overexpressed in cancerous hepatocytes from HCC; two antigens, HBV core antigen and HBV DNA polymerase, which are associated with chronic HBV infection. Each peptide contained 20–40 amino acids with one or several formerly identified T cell epitopes (Additional file 1: Table S1). These synthesized long peptides could be processed into epitopes by mature DCs, presented on their surfaces, and further recognized by T cells with diverse HLA genotypes. Therefore, the HLA genotypes of the 13 patients treated with MASCT were not previously screened and selected.

In total, 71 injections of DCs and 100 injections of activated T cells were received by the 13 patients. Only five adverse events (grade 1) were recorded after DC injection and 12 adverse events (8 of grade 1, 3 of grade 2, and 1 of grade 3) were observed after activated T cell injection (Table 2). We also investigated the characteristics of the 13 patients during MASCT. No significant change was observed (Additional file 1: Table S3). These data suggested that MASCT was well-tolerated by our patients.

**Table 2:** Adverse events during MASCT treatment

Adverse events	DC injection					T cell injection				
	Treatment times (n = 71)				Patients	Treatment times (n = 100)				Patients
	Grade 1 (%)	Grade 2 (%)	Grade 3 (%)	Grade 4 (%)	n = 13 (%)	Grade 1 (%)	Grade 2 (%)	Grade 3 (%)	Grade 4 (%)	n = 13 (%)
Constitutional symptoms										
Fever	2 (2.8)	0 (0)	0 (0)	0 (0)	2 (15.4)	1 (1)	0 (0)	0 (0)	0 (0)	1 (7.7)
Musculoskeletal disorders										
Osteodynia	1 (1.4)	0 (0)	0 (0)	0 (0)	1 (7.7)	0 (0)	0 (0)	0 (0)	0 (0)	0 (0)
Respiratory tract disorders										
Cough	1 (1.4)	0 (0)	0 (0)	0 (0)	1 (7.7)	3 (3)	0 (0)	0 (0)	0 (0)	2 (15.4)
Throat	1 (1.4)	0 (0)	0 (0)	0 (0)	1 (7.7)	3 (3)	0 (0)	0 (0)	0 (0)	2 (15.4)
Blood examinations										
White blood cell decrease	0 (0)	0 (0)	0 (0)	0 (0)	0 (0)	0 (0)	1 (1)	0 (0)	0 (0)	1 (7.7)
Platelet decrease	0 (0)	0 (0)	0 (0)	0 (0)	0 (0)	0 (0)	2 (2)	0 (0)	0 (0)	2 (15.4)
Neutrophil decrease	0 (0)	0 (0)	0 (0)	0 (0)	0 (0)	1 (1)	0 (0)	0 (0)	0 (0)	1 (7.7)
Hemoglobin decrease	0 (0)	0 (0)	0 (0)	0 (0)	0 (0)	0 (0)	0 (0)	1 (1)	0 (0)	1 (7.7)
Immune diseases										
Allergic reaction	2 (2.8)	0 (0)	0 (0)	0 (0)	2 (15.4)	3 (3)	0 (0)	0 (0)	0 (0)	2 (15.4)

The grades of adverse events are defined according to common terminology criteria for adverse events (CTCAE)

### Immunological characteristics of DCs and activated T cells in MASCT

**Fig. 2:**
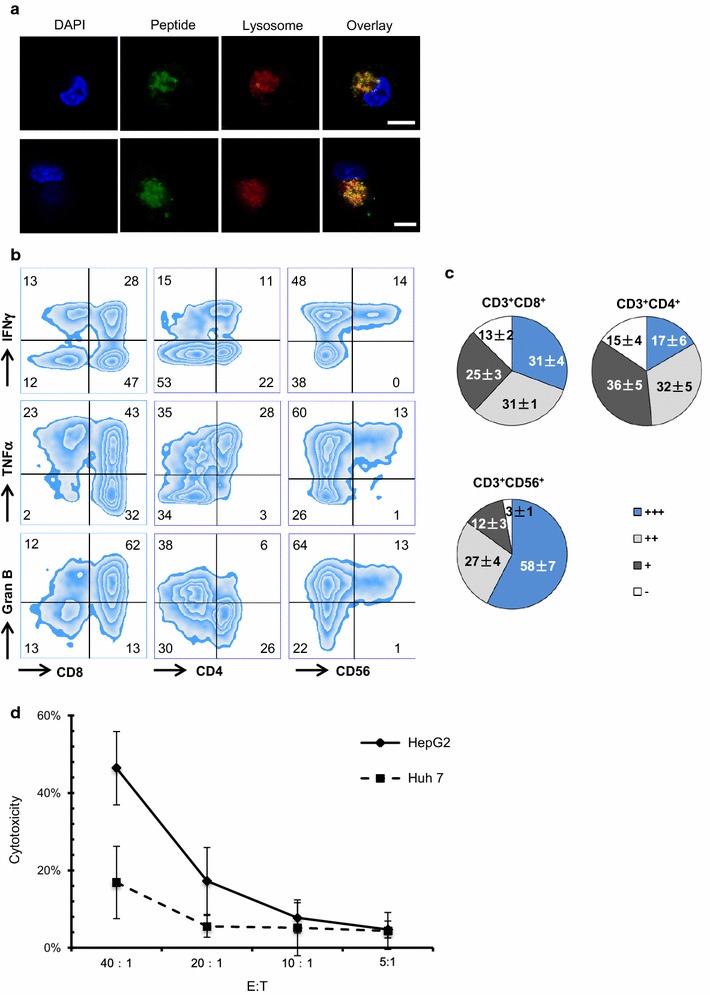
The characteristics of mature DCs and activated T cells served in MASCT treatment. **a** The cellular uptake of peptides by immature DCs. Human monocyte-derived immature DCs were pulsed with fluorescent-labeled peptide of survivin (*green*, 2.5 μg/mL) for 2 h, followed by labeling with DAPI (*blue*) and lysotracker (*red*) to identify the nuclei and lysosomes, respectively. Fluorescent images were recorded by confocal microscopy, and the images are representative of four independent experiments. The *scale bar* is 7.5 μm. **b** The intracellular production of IFNγ, TNFα and granzyme B in the subsets of CD3^+^CD8^+^ T cells, CD3^+^CD4^+^ T cells, and CD3^+^CD56^+^ T cells. Activated T cells generated from HCC patients were stimulated with PMA for 4 h before being examined by flow cytometry. **c** Pie charts displaying the percentage of T cell subsets that co-expressed cytokines and enzymes. The mean ± SEM was shown. Triple producers: *blue*; double producers: *gray*; single producers: *dark*; non-producer: *white*. **d** Activated T cells generated from HLA-A2^+^ patients exhibited greater cytotoxic activity against the HCC cell line HepG2 (HLA-A2^+^) than HuH-7 cells (HLA-A2^−^) at different E:T ratios (the ratio of effector cells to target cells), such as 40:1, 20:1, 10:1, and 5:1

The average amount of DCs received by patients was 2.23 × 10^7^. Full immune functional properties are shown by the high expression of maturation signature molecules on the surface of DCs such as HLA-DR, CD86, CD80, and CD83 (Table 3A; Additional file 1: Figure S1A). Moreover, these DCs secreted high level of pro-inflammatory cytokine, IL12, but low levels of immunosuppressive cytokine, IL10 (Additional file 1: Figure S1B). Particularly, immunofluorescence experiments demonstrated that long peptides could be effectively internalized by the immature DCs and were primarily localized in the cytosol instead of the lysosomes (Fig. 2a), which consequently promoted cross-presentation by MHC I molecules and stimulation of CD8^+^ cytotoxic T cells.

**Table 3:** The counts and immunological profiles of mature DCs (A) and activatedT cell (B) in MASCT

	Total counts (×10^9^)	HLA-DR^+^ (%)	CD80^+^ (%)	CD86^+^ (%)	CD83^+^ (%)		
A							
Mean ± SD	2.23 ± 1.21	90.85 ± 8.19	88.97 ± 15.60	89.35 ± 11.29	67.91 ± 20.52		

	Total counts (×10^9^)	CD3^+^ (%)	CD8^+^ (%)	CD4^+^ (%)	CD3^+^CD56^+^ (%)	CD8^+^CD107a^+^ (%)	Tregs (%)
B							
Mean ± SD	6.63 ± 2.11	92.7 ± 5.26	56.5 ± 16.90	29.28 + 16.16	13.63 ± 12.60	50.31 ± 16.28	0.10 ± 0.20

The regulatory T cells (Tregs) were defined as CD4^+^CD25^+^FoxP3^+^ expression

After co-culturing with DCs pulsed with the peptide pool, the activated T cells proliferated to the average amount of 6.63 × 10^9^ cells (Table 3B). The cells were almost exclusively CD3^+^, with a major percentage being CD3^+^CD8^+^ cells and a minor proportion being CD3^+^CD4^+^ and CD3^+^CD56^+^ cells, but an insignificant amount of regulatory T cells (Treg) was detected (Table 3B; Additional file 1: Figure S2A). Moreover, pro-inflammatory cytokines such as IFNγ and TNFα were detected in the supernatants of cultured activated T cells, while the immune inhibitory cytokines IL10 and IL4, were hardly detected (Additional file 1: Figure S2B). Further analysis of activated T cells revealed a poly-functional property in the major subset of CD3^+^CD8^+^ cytotoxic T cells and in the minor subsets of CD3^+^CD4^+^ helper T cells and CD3^+^CD56^+^ NKT cells, characterized by the co-expression of considerable amounts of IFNγ, TNFα, and granzyme B (Fig. 2b, c), as compared to the non-activated T cells isolated from the same patients (Additional file 1: Figure S2C). Besides, the activated T cells generated from HLA-A2^+^ patients exhibited superior cytotoxic activity against the HLA-A2^+^ HCC cell line, HepG2, than against the HLA-A2^−^ HCC cell line Huh-7 cells (Fig. 2d), suggesting an HLA-restricted killing capacity.

### MASCT elicited antigen-specific immune responses in patients with HCC

**Fig. 3:**
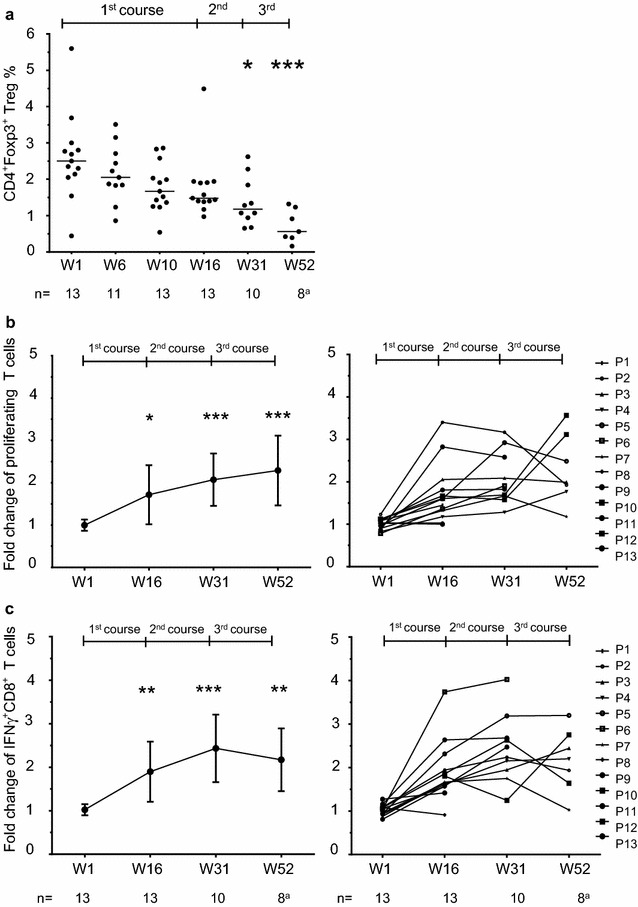
The improvement of immune response in HCC patients during repeated courses of MASCT treatment. **a** The significant decrease of the frequency of Tregs in HCC patients’ PBMCs during three courses of MASCT treatment. The antigen-specific proliferation of T cells (**b**) and intracellular IFNγ production of CD8^+^ T cells (**c**) in HCC patients’ PBMCs were raised during three courses of MASCT treatment. The fold changes of frequency of proliferating of T cells or IFNγ production of CD8^+^ T cells were analyzed by comparing patients’ PBMCs stimulated by multiple antigen peptide pool with PBMCs stimulated by irrelevant peptide. The results representing the overall review of 13 patients were shown in mean ± SD on the *left panel*, and the graphics demonstrating antigen-specific immune responses of each patient were on the *right panel*. ^a^ Eight patients were tumor-free on week 52, however, one PBMCs sample on week 52 (No. 6) was damaged during thawing. One-way ANOVA test was used for statistical analysis. *p < 0.05, ** *p* < 0.01, *** *p* < 0.001

To investigate whether MASCT could improve the immune environment in patients with HCC, we examined the immune profiles of PBMCs from the patients during MASCT. No significant change was detected in most of the immune cell subpopulations (Additional file 1: Table S4). However, we observed a continuous reduction of Tregs during the repeated MASCT (Fig. 3a). This decrease became significant after two courses of MASCT (week 31, *p* = 0.014), and even more significant after three courses of MASCT (week 52, *p* < 0.001). Furthermore, proliferation assay (Fig. 3b) and IFNγ production assay (Fig. 3c; Additional file 1: Figure S3B) were performed to investigate antigen-specific T cell responses by stimulating patients’ PBMCs with the antigen peptide pool. As expected, increased antigen-specific T cell proliferation and IFNγ production were observed in patients’ PBMCs during MASCT. Significantly enhanced antigen specific immune responses were detected after the first course of MASCT (week 16, proliferation: *p* = 0.01; IFNγ production: *p* = 0.003), and became even stronger after the second course (week 31, proliferation: *p* < 0.001; IFNγ production: *p* < 0.001). Notably, these antigen-specific immune responses induced by MASCT were maintained until week 52 (proliferation: *p* < 0.001; IFNγ production: *p* = 0.001), which was 2 months after the end of the three courses of MASCT (Fig. 3b, c). Interestingly, the antigen-specific CD8^+^ T cells producing high levels of IFNγ also co-expressed CD27 and CD28 on their surfaces (Additional file 1: Figure S3A), which indicated the potential to acquire an immune memory phenotype [2, 25].

Thus, in MASCT-treated patients, both down-regulation of Treg and up-regulation of antigen-specific T cells were detected, demonstrating that MASCT improved the immune environment and reinforced the immune responses in patients with HCC.

### MASCT-induced immune responses against each antigen peptide in the pool

**Fig. 4:**
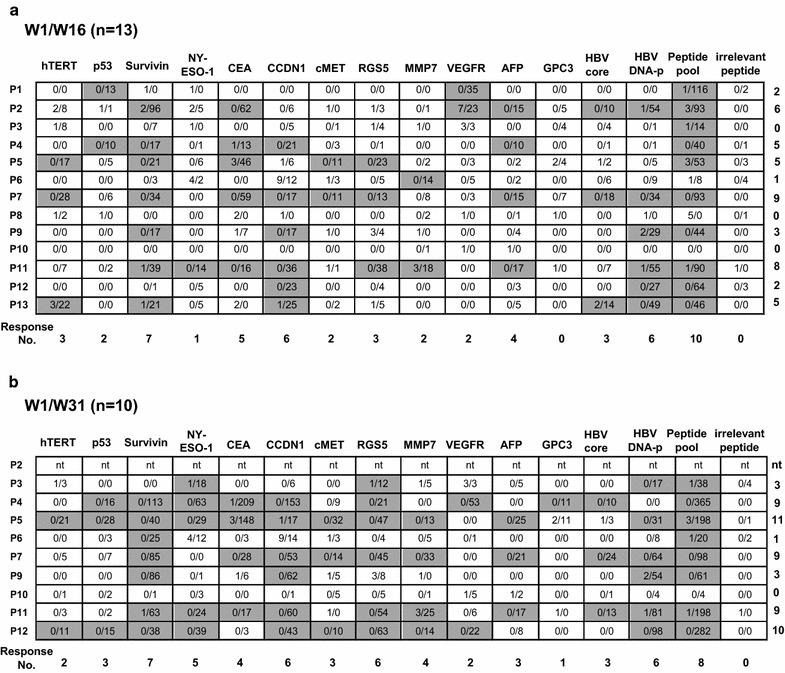
The dynamic immune responses against each kind of antigen were induced by MASCT. The specific immune responses of HCC patients’ PBMCs against each kind of antigens were detected by IFNγ ELISPOT assay. **a** The spots of IFNγ secreting T cells on the time point of week 1 (baseline) and week 16 (after the first course of MASCT treatment) were counted and compared. **b** The spots of IFNγ secreting T cells on the time point of week 1 (baseline) and week 31 (after the second course of MASCT treatment) were counted and compared. The shown *numbers* indicated the IFNγ^+^ spots numbers in 200,000 cells, and were normalized by minus the IFNγ^+^ spots numbers of the PBMCs stimulated with irrelevant peptide. The clear MASCT-induced antigen-specific immune responses were marked in *grey* as defined by at least ten more spots than baseline

Although MASCT-induced immune responses against the antigen peptide pool were observed, we investigated which antigens contributed to these immune responses. To answer this question, specific immune responses against each antigen were further examined by IFNγ ELISPOT assay at week 1 (baseline), week 16 (end of the first course of MASCT), and week 31 (end of the second course of MASCT) in patients’ PBMCs. All 13 patients finished the first course of MASCT, and clear antigen-specific immune responses were detected in 11 patients’ PBMCs (except patients Nos. 8 and 10) as compared to baseline. However, the patterns of immune responses varied from patient to patient. Patient No. 7 was the best immunologically responding patient, showing specific immune responses against nine different antigens out of fourteen in total, while the worst responding patient, patient No. 6, presented specific responses against only one antigen (Fig. 4a; Additional file 1: Figures S4, S5). Ten patients were further tested for immune responses after the second course of MASCT since they were still diagnosed as tumor-free. Generally, the overall antigen-specific immune responses of the ten patients grew stronger and covered more antigens (Fig. 4b; Additional file 1: Figures S4, S5). The best immunologically responding patient was patient No. 5, who responded to more antigens than after the first course of MASCT, showing an increase from 5 to 11 antigens. Patient No. 7 still presented a good response against nine antigens; however, the type of antigens had changed. The antigen-specific response against hTERT, which was remarkable on week 16, became nearly undetectable on week 31, while antigen-specific response against MMP7 appeared on week 31. Besides, the antigen-specific responses against survivin, CCDN1, cMET, RGS5, AFP, HBV core antigen, and HBV DNA polymerase were enhanced, while the response against CEA was slightly reduced. This change was observed in all ten patients analyzed on week 31 (Fig. 4a, b; Additional file 1: Figures S4, S5), suggesting that immunological response patterns to MASCT were dynamic in individual patient.

The most immunogenic antigen was survivin, which induced specific immune responses in seven patients out of 13 (7/13). Moreover, the immunological responses against CCND1 (6/13), RGS5 (6/13), CEA (5/13), HBV DNA polymerase (5/13), MMP7 (4/13), and AFP (4/13) were good. However, few patients responded to hTERT (3/13), p53 (3/13), cMET (3/13), HBV core antigen (3/13), VEGFR (2/6), and GPC3 (1/6) (Fig. 4a, b; Additional file 1: Figures S4, S5).

Thus, dynamic and individual antigen-specific antigen responses elicited by MASCT indicated the advantages and requirements to treat patients with multiple antigens in cell-based cancer immunotherapies instead of a single target. The stronger and wider immune responses against multiple antigens on week 31 also indicated the benefits of repeated MASCT.

### Improvement of MASCT-induced immune responses may correlate with clinical outcomes

**Fig. 5:**
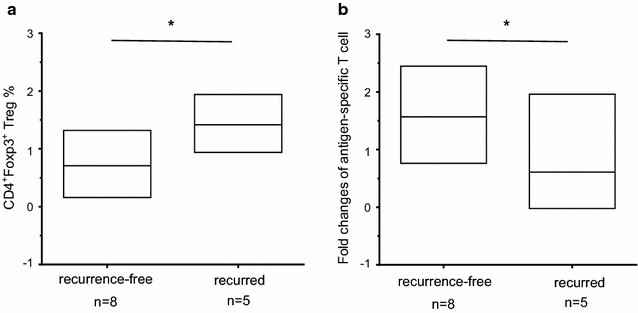
The improvement of MASCT-induced immune responses in tumor recurrence-free HCC patients. **a** The frequency of Tregs in tumor recurrence-free HCC patients’ PBMCs was lower than that in tumor recurred patients. **b** The MASCT-induced antigen peptide pool-specific immune responses in tumor recurrence-free patients were stronger than the immune responses in patients with tumor recurrence. The fold changes of antigen-specific proliferating of T cells were analyzed by comparing patients’ PBMCs stimulated by multiple antigen peptide pool with PBMCs stimulated by irrelevant peptides. The data collected on the last time point were used for the analysis. The results were shown in mean ± SD. Two-sample t test was used for statistical analysis. *p < 0.05

As described above, 13 HCC patients with total tumor removal received repeated MASCT, during which no other treatment was applied. Patients were routinely checked by CT scan to detect tumor recurrence every 13 weeks during 52 weeks in total. Based on the CT scan results, these 13 patients were divided into two groups, the tumor recurrence-free patients (n = 8) and the patients presenting with recurrence (n = 5). The frequency of Tregs in tumor recurrence-free patients’ PBMCs was significantly lower (*p* = 0.015) than that in PBMCs from patients presenting with recurrence (Fig. 5a). The specific immune responses against multiple antigen peptide pool were also compared between the two groups. Although no clear difference of the antigen peptide pool induced IFNγ production was discovered between patients from the two groups, a significantly better antigen peptide pool-specific T cell proliferation (*p* = 0.037) was observed in the recurrence-free patients (Fig. 5b), indicating that stronger MASCT-induced antigen-specific immune responses may correlate with better clinical outcomes of patients with HCC.

## Discussion

Currently, cell-based cancer immunotherapies are well accepted as effective alternative therapies for cancer patients, particularly for those who did not respond to conventional cancer therapies such as surgery, radiation therapies, chemotherapies, and targeted therapies. However, cell-based cancer immunotherapies still present great challenges. Ex vivo generated DCs loaded with different forms of tumor antigens such as tumor lysates, tumor total mRNA, tumor proteins, or peptides were injected into cancer patients as therapeutic vaccines. However, whether DC vaccines could defeat the immune suppressive tumor microenvironment and efficiently activate and expand tumor-specific CD8^+^ and CD4^+^ T cells, which could further eliminate tumor cells, remained questionable [2]. After infiltrating into the tumor microenvironment, tumor-specific T cells recognize tumor cells presenting particular tumor antigens and release cytotoxic factors to lyse them. Unfortunately, numerous immune inhibitory cells such as Treg and myeloid-derived suppressor cells (MDSCs) are also present in the tumor microenvironment. They secrete massive immunosuppressive factors, which shut down the immune surveillance by causing the dysfunction of tumor-specific effector T cells as well as the loss of expression or mutation of antigen in tumor cells. The expression of PD-L1 on the surface of tumor cells also contributes to the suppression of tumor-specific T cells. Although genetically modified T cells with T cell receptors (TCRs) or CARs may conquer the central and peripheral immune tolerance of tumor antigens [26], the treatment still failed instantly when the tumor cells lost the expression of the specific epitope targeted by TCRs or CARs.

To our knowledge, MASCT is the first application to combine DC vaccine and adoptive T cell transfer in one treatment, although the strategy to activate and amplify tumor-specific T cells out of the autologous T cell repertoire is no longer considered cutting edge. Cancer patients received injections of DCs pulsed with multiple tumor antigens as well as activated T cells stimulated with these DCs, which not only helped to generate tumor-specific T cells in vivo, but also allowed us to directly transplant ex vivo prepared tumor-specific T cells to cancer patients. The adoptively infused tumor-specific T cells could also be continuously activated by the previously injected DCs. Moreover, in this study, we demonstrated that T cells specifically responding to different antigens were successfully elicited in patients with HCC at the same time, which may more effectively prevent the immune escape caused by the loss or mutation of a single tumor antigen. Individual and dynamic T cell responding patterns were observed in patients during MASCT, which may be the consequence of the diversity in term of antigen expression and presentation in tumor cells, the potential of T cell repertoire, and the variability of the tumor microenvironment. The most immunogenic tumor antigen for the 13 patients with HCC enrolled in this study was survivin, a well-known oncogene, which negatively regulates cell apoptosis by inhibiting caspase activation. Another tumor antigen, CCND1, which is associated with cell cycle, also induced stronger immune responses in patients with HCC. Deceivingly, the HCC specific oncogene, GPC3, which is often used as the target in immunotherapies for HCC [23, 24, 27], only induced immune responses in one patient after two courses of MASCT. Chronic virus infection induces severe immune tolerance in patients, which accounts for the problems of virus clearance. However, in this study, specific responses against HBV DNA polymerase (5/13) and HBV (3/13) core antigen were successfully restored in some of the patients with HCC, which may benefit from the treatment with a DC vaccine that reprogrammed the tolerant HBV-specific memory T cells. Thus, using multiple antigens in MASCT to target tumor cells instead of a single tumor antigen is a more promising approach. The approach to treat cancer patients with antigen loaded DC vaccine or amplified tumor-specific T cells from the autologous T cell repertoires may not be novel. However, to our knowledge, MASCT is the first strategy to combine DC vaccine and ACT, and to manufacture DCs with multiple tumor antigens, both of which made MASCT an innovative cell-based cancer immunotherapy.

We used synthetic long antigen peptides rather than short epitope peptides in MASCT. Each peptide was 20–40 amino acid residues, which facilitated the presentation of epitopes applicable for various HLA genotypes. Based on this mechanism, we did not screen the HLA genotypes of patients with HCC and selected a particular HLA genotype matching the tumor antigen epitopes in this study. MASCT was applied to patients with HCC without HLA restriction and still successfully induced antigen-specific immune responses in all patients.

HCC is a type of cancer with high morbidity, especially in males. One-tenth of the population in China is chronically infected with HBV. Infected individuals present high risks of developing HCC later in their lives. With few effective therapies, HCC also presents the leading mortality. In an attempt to solve this medical need, we applied MASCT in patients with HCC after tumor resection, aiming at preventing cancer recurrence. In this study, 13 patients with HCC received repeated MASCT after tumor resection. Five of them presented with tumor recurrence within 52 weeks during or after finishing MASCT. Notably, the specific immune responses against the peptide pool of multiple antigens in these five patients were significantly weaker than those in the other eight tumor-free patients. This observation indicated that patients who better responded to MASCT presented with better clinical outcomes, which correlated with a previous result demonstrating that T cell responses to human papillomavirus (HPV) 16 and 18 E6 protein in patients with cervical intraepithelial neoplasia treated with a therapeutic synthetic DNA vaccine correlated with histopathological regression and viral clearance [28]. However, we were unable to identify a specific or several antigens dominating the immune responses that would account for the correlation with clinical benefits. Although some candidates such as survivin and RGS5 were highlighted, no conclusive results were obtained probably due to the small sample size in this study. Future studies are warranted in which a higher number of patients with HCC presenting with various cancer stages will receive MASCT.

There are still limitations of this study since we have only applied MASCT to patients with HCC after resection. The mechanism and safety of MASCT in tumor bearing patients need to be further investigated. We also plan to adjust the components of the multiple antigen peptide pool based on the expression of antigens in the patients’ tumors.

## Conclusions

Our study is the first to demonstrate that specific responses of T cells against multiple antigens can be strongly induced and increased in vivo by MASCT and that MASCT is well-tolerated by patients with HCC. The same principle and methodology are being explored for other tumors. Furthermore, we speculate that MASCT can be combined with immune checkpoint blockade therapy such as anti-PD1 antibody. Given the fact that immune checkpoint blockade therapy only brings clinical benefits to less than 20% cancer patients, the most non-responding patients may not have enough pre-existing tumor specific T cells, which may be rescued by MASCT.

## Additional file

**Additional file 1.** Additional figures and tables.

## Abbreviations

MASCT: multiple antigens stimulating cellular therapy
HCC: hepatocellular carcinoma
TAA: tumor associated antigen
PBMCs: peripheral blood mononuclear cells
DCs: dendritic cells
ACT: adoptive cell therapy
TIL: tumor-infiltrating lymphocytes
TCRs: T cell receptors
CARs: chimeric antibody receptors
HBV: hepatitis B virus
TACE: transcatheter arterial chemoembolization
BCLC: Barcelona Clinic Liver Cancer
CT: computed tomography
Treg: regulatory T cells
MDSCs: myeloid-derived suppressor cells
HPV: human papillomavirus
