# Supplementary material for: Dynamic and specific immune responses against multiple tumor antigens were elicited in patients with hepatocellular carcinoma after cell-based immunotherapy
Source: J Transl Med. 2017 Mar 22;15:64. doi: 10.1186/s12967-017-1165-0 (PMC5363021; doi:10.1186/s12967-017-1165-0)
Supplement: Supplementary file 1 — Additional file 1. Additional figures and tables. [file 12967_2017_1165_MOESM1_ESM.zip › Figure S3.pptx]

## Slide 1
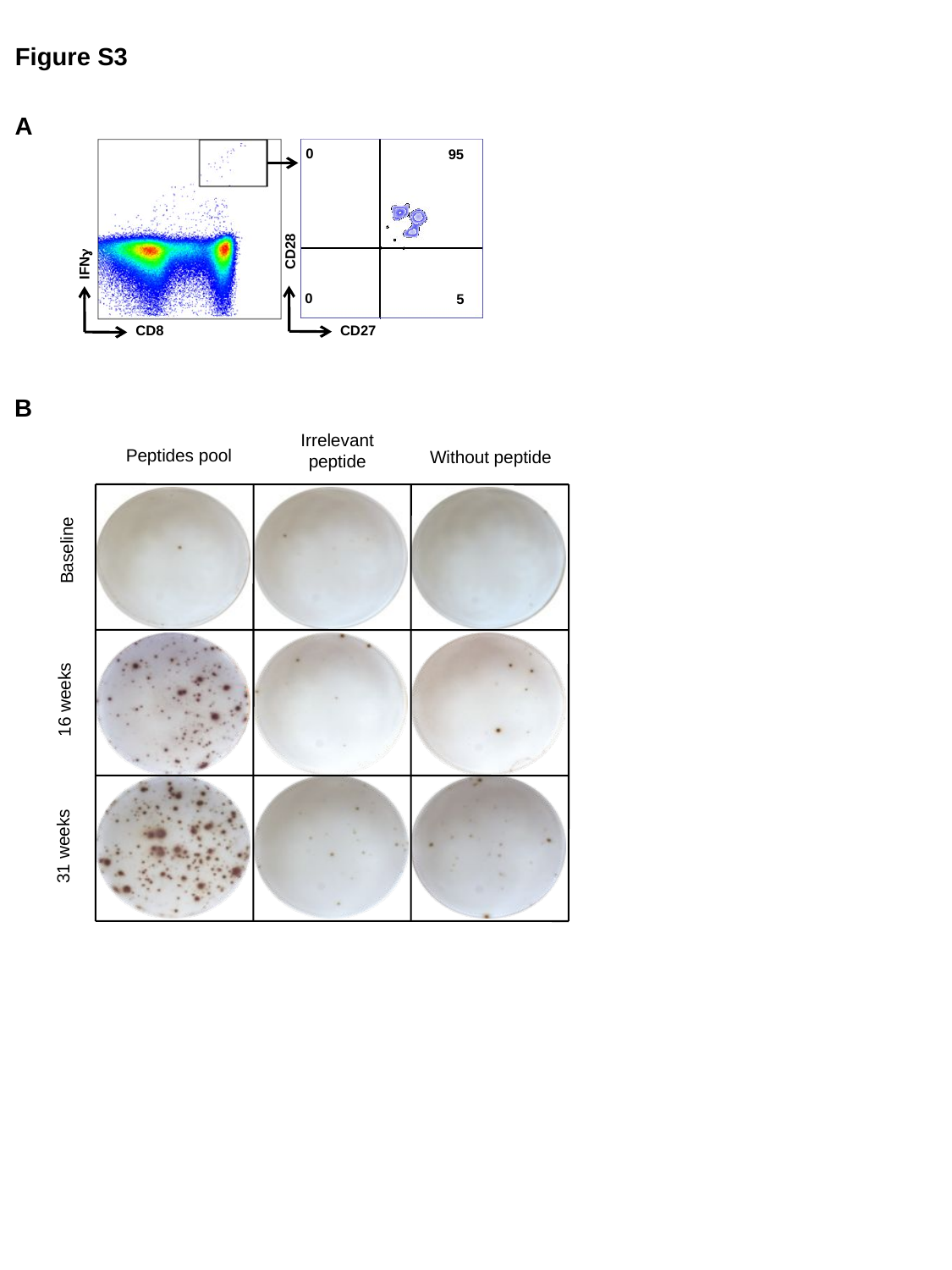

Figure S3
A
 0
95
CD28
IFNγ
 0
 5
CD27
CD8
B
Irrelevant peptide
Peptides pool
Without peptide
Baseline
16 weeks
31 weeks
