# Supplementary figures and images for: Dynamic and specific immune responses against multiple tumor antigens were elicited in patients with hepatocellular carcinoma after cell-based immunotherapy
Source: J Transl Med. 2017 Mar 22;15:64. doi: 10.1186/s12967-017-1165-0 (PMC5363021; doi:10.1186/s12967-017-1165-0)

## Slide 1
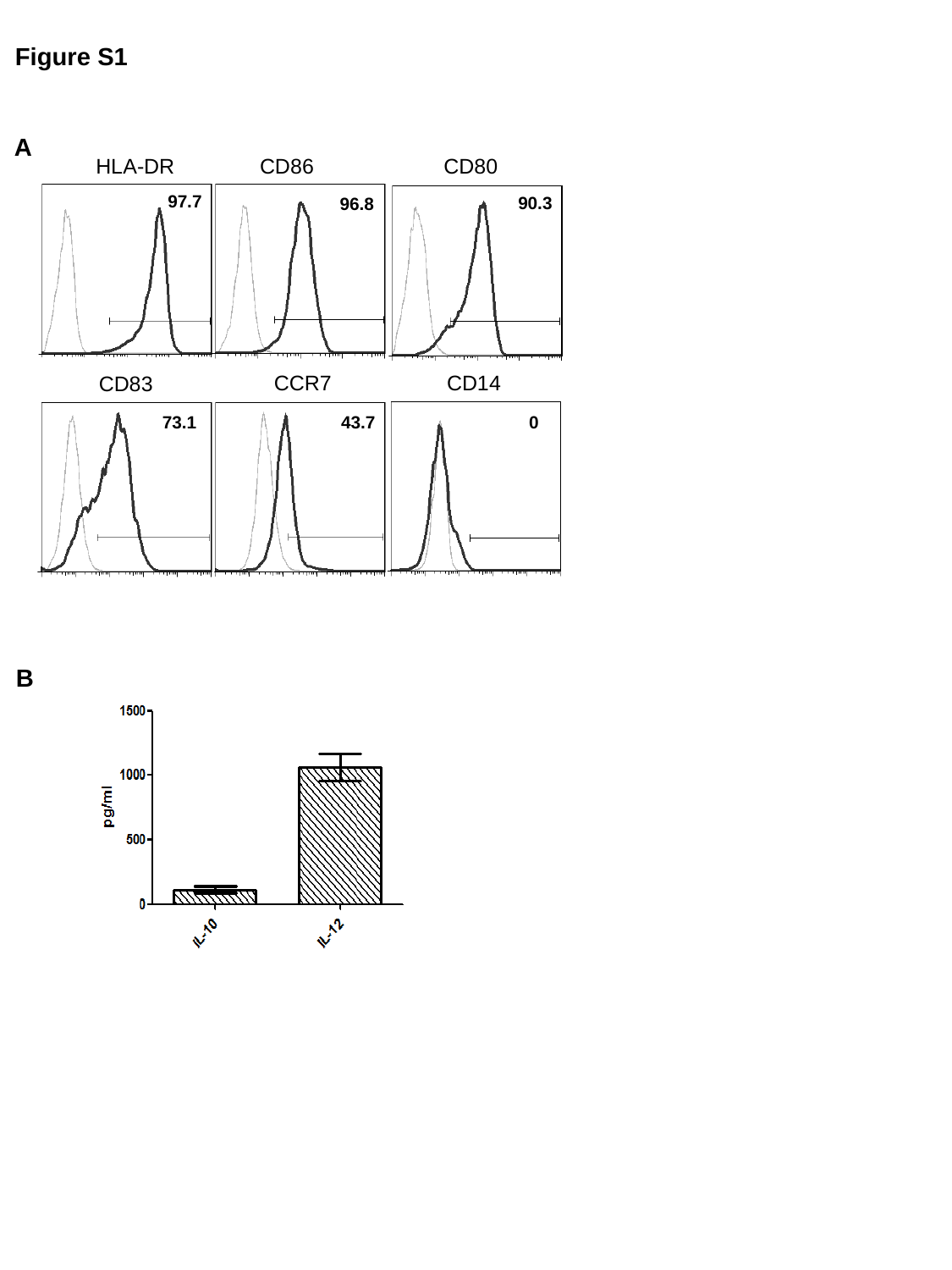

Figure S1
A
HLA-DR
CD80
CD86
97.7
90.3
96.8
CCR7
CD14
CD83
73.1
43.7
0
B

Supplement: Supplementary file 1 — Additional file 1. Additional figures and tables. [file 12967_2017_1165_MOESM1_ESM.zip › Figure S1.pptx]

## Slide 1
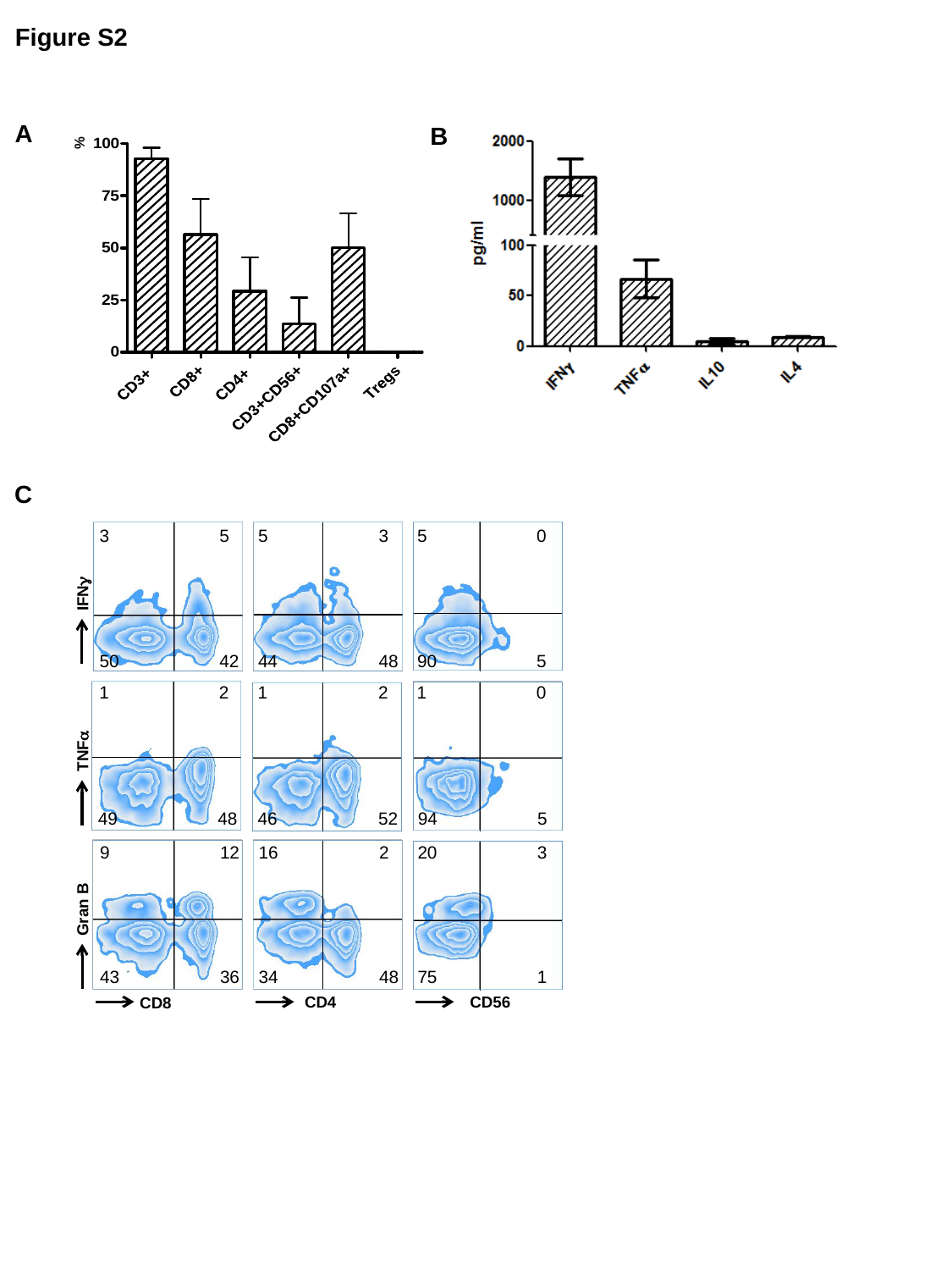

Figure S2
%
A
B
C
3
5
5
3
5
0
IFNg
50
42
44
48
90
5
1
2
1
2
1
0
TNFa
49
48
46
52
94
5
9
12
16
2
20
3
Gran B
43
36
34
48
75
1
CD4
CD56
CD8

Supplement: Supplementary file 1 — Additional file 1. Additional figures and tables. [file 12967_2017_1165_MOESM1_ESM.zip › Figure S2.pptx]

## Slide 1
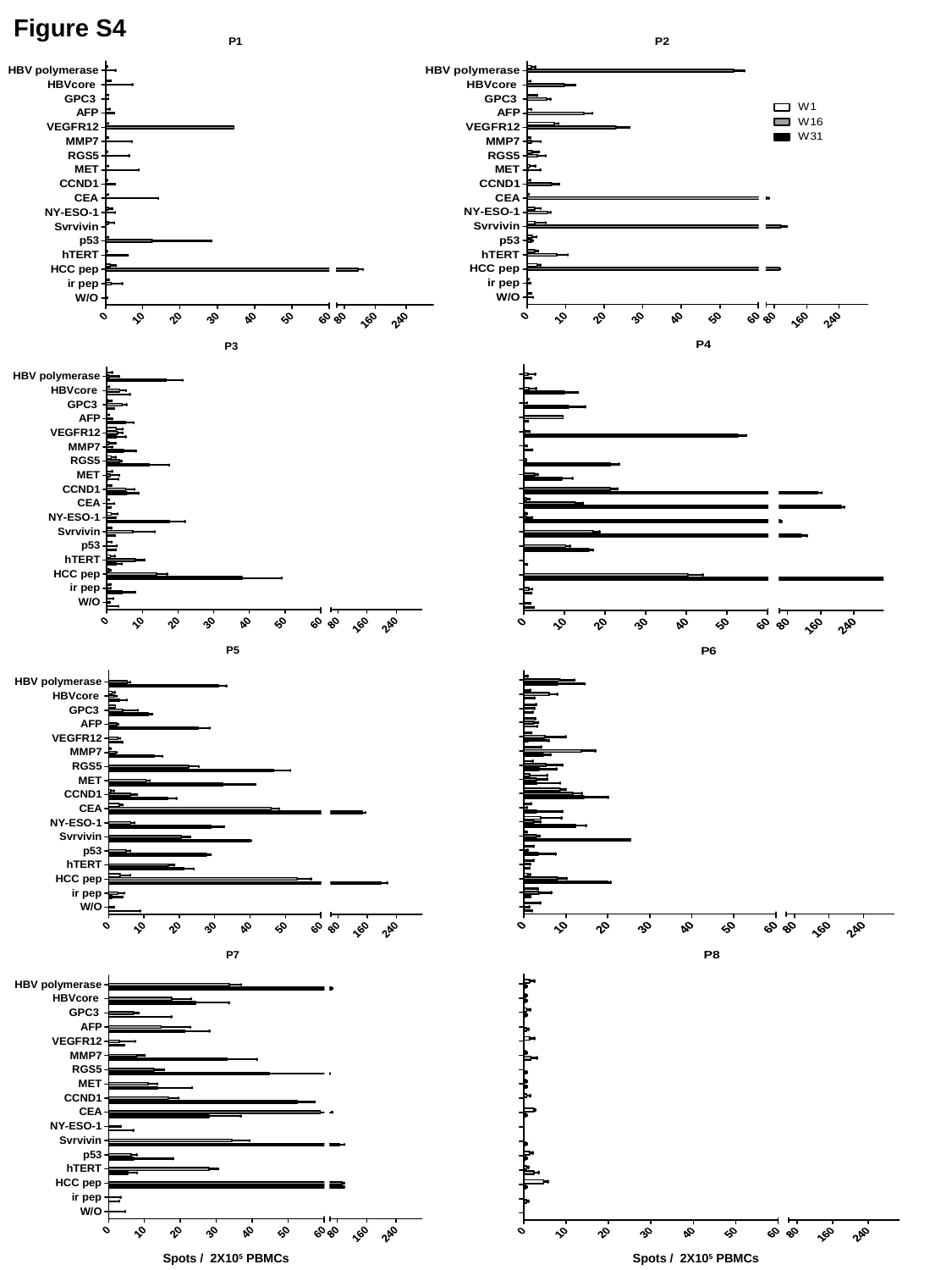

Figure S4
Spots / 2X105 PBMCs
Spots / 2X105 PBMCs

Supplement: Supplementary file 1 — Additional file 1. Additional figures and tables. [file 12967_2017_1165_MOESM1_ESM.zip › Figure S4.pptx]

## Slide 1
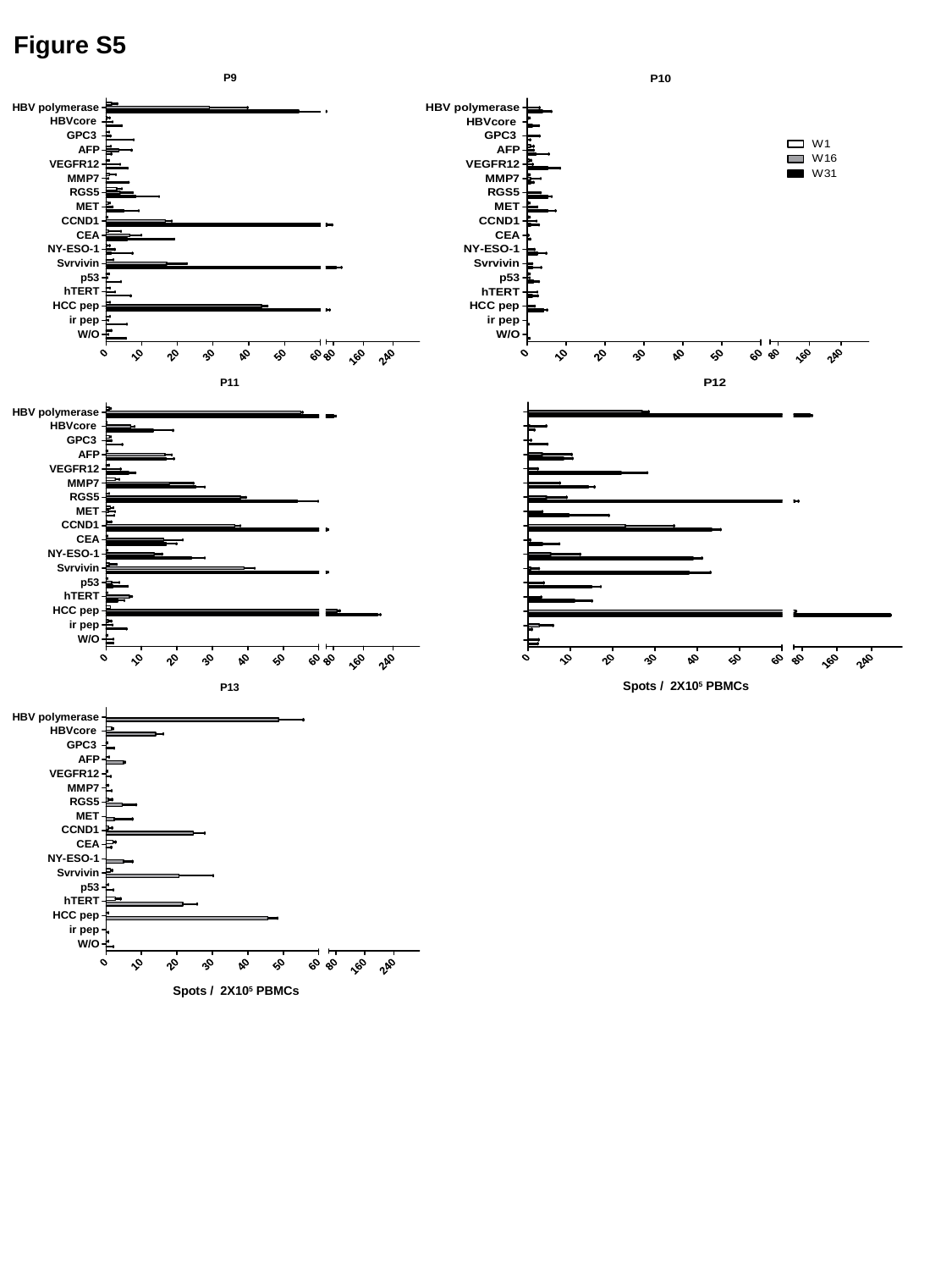

Figure S5
Spots / 2X105 PBMCs
Spots / 2X105 PBMCs

Supplement: Supplementary file 1 — Additional file 1. Additional figures and tables. [file 12967_2017_1165_MOESM1_ESM.zip › Figure S5.pptx]
