# Supplementary material for: Dynamic and specific immune responses against multiple tumor antigens were elicited in patients with hepatocellular carcinoma after cell-based immunotherapy
Source: J Transl Med. 2017 Mar 22;15:64. doi: 10.1186/s12967-017-1165-0 (PMC5363021; doi:10.1186/s12967-017-1165-0)
Supplement: Supplementary file 1 — Additional file 1. Additional figures and tables. [file 12967_2017_1165_MOESM1_ESM.zip › Supplementary Materials revised.doc]

**Supplementary Materials:**

**Supplemental figure legends:**

**Fig. S1. The immunological characteristics of mature DCs.** (A) Mature DCs expressed high level of MHC molecule (HLA-DR), co-stimulatory molecules (CD80, CD83 and CD86) and chemokine receptor (CCR7), but loosed the expression of CD14 totally. The histograms showed the expression of these molecules on the surface of mature DCs (black), as compared to immature DCs (gray). (B) Mature DCs secret high level of pro-inflammatory cytokine IL12, but low level of inhibitory cytokine IL10.

**Fig. S2. The characteristics of the T cells generated from HCC patients with or without *ex vivo* stimulation.** (A) The immune profiless of activated T cells generated from HCC patients. Mean ± SD of was shown. (B) The activated T cells generated from HCC patients secreted significant amount of IFNγ and TNFα but no IL10 and IL4. Mean± SD was shown. (C) T cells isolated from HCC patients were stimulated with PMA for 4 hours. Flow cytometry was used to detect the intracellular production of IFNγ, TNFα and Granzyme B in the subets of CD3^+^CD8^+^, CD3^+^CD4^+^ and CD3^+^CD56^+^ T cells.

**Fig. S3. The immune responses were raised in the HCC patients during MASCT treatment.** (A) The antigen-specific CD8^+^ T cells producing high amount of IFNγ also co-expressed the memory marker CD27 and CD28. (B) Photos of the IFNγ^+^ spots in wells of an ELISPOT plate. Patient`s PBMCs stimulated by antigen peptide pool, irrelevant peptide, and PBMCs without stimulation on the time points of baseline, week 16 and week 31 were shown.

**Fig. S4-5. The IFNγ^+^ ELISPOT assay results of each patient.** IFNγ^+^ ELISPOT assays were performed in thirteen patients by stimulating patients` PBMCs by each kind of antigen peptide, antigen peptide pool, and irrelevant peptide on baseline, week 16 and week 31. Spots per 2X10^5^ PBMCs were shown.

Table S1. The epitopes contained in the peptides pool.

| No. | Antigen | Description | Containing epitopes |
| --- | --- | --- | --- |
| 1 | hTERT | human Telomerase Reverse Transcriptase | VYAETKHFL, VYGFVRACL, ILAKFLHWL, LTDLQPYMR, DLQVNSLQTV |
| 2 | p53 | Tumor suppressing antigen | STPPPGTRV，AIYKQSQHM, EYLDDRNTF, LLGRNSFEVRV |
| 3 | Survivin | Apoptosis inhibitor | RISTFKNWPFL, DLAQCFFCFK, ELTLGEFLKL |
| 4 | NY-ESO-1 | Tumor-testis antigen | YLAMPFATPM, SLLMWITQC |
| 5 | CEA | Carcinoembryonic Antigen | YLSGANLNL, TYACFVSNL |
| 6 | CCND1 | Cyclin D1 | LLGATCMFV |
| 7 | MET | HGF-hepatocyte growth factor receptor | YVDPVITSI |
| 8 | RGS5 | Regulators of G protein Signaling 5 | LAALPHSCL |
| 9 | MMP7 | Matrix Metalloproteinase 7 | SQDDIKGIQK |
| 10 | VEGFR | Vascular Endothelial Growth Factor Receptor | TLFWLLLTL, SYGVLLWEI, RFVPDGNRI, SYMISYAGM |
| 11 | AFP | Alpha Fetoprotein | PLFQVPEPV，FMNKFIYEI, GLSPNLNRF, GVALQTMKQ |
| 12 | GPC3 | Glypican-3 | FVGEFFTD, EYILSLEEL |
| 13 | HBVc | HBV core antigen | FLPSDFFPS, YVNVNMGLK |
| 14 | HBVp | HBV DNA polymerase | GLSRYVARL, FLLSLGIHL, RKYTSFPWLL |

**Table S2. Characteristics of HCC patients at baseline**

| Characteristics | P1 | P2 | P3 | P4 | P5 | P6 | P7 | P8 | P9 | P10 | P11 | P12 | P13 |
| --- | --- | --- | --- | --- | --- | --- | --- | --- | --- | --- | --- | --- | --- |
| Sex, male/female (M/F) | F | M | M | M | F | M | M | F | M | M | M | M | F |
| Age, years | 61 | 45 | 61 | 39 | 34 | 35 | 46 | 61 | 23 | 28 | 46 | 30 | 41 |
| CEA, ng/mL | 0.88 | 2.94 | 4.91 | 2.41 | 1.51 | 1.40 | 1.83 | 1.89 | 3.63 | 8.94 | 4.97 | 0.09 | 0.82 |
| AFP, ng/mL | 2.67 | 5.68 | 4.19 | 11.45 | 7.58 | 5.01 | 13.43 | 55.30 | 24.10 | 6.45 | 5.04 | 2.2 | 10.47 |
| ALT, ng/mL | 22 | 23 | 15 | 32 | 18 | 161 | 41 | 29 | 35 | 47 | 18 | 19 | 12 |
| AST, ng/ml | 20 | 28 | 33 | 30 | 23 | 66 | 33 | 3 | 30 | 62 | 18 | 26 | 18 |
| TIBL, ng/mL | 8.9 | 6.5 | 10.2 | 8.2 | 6.9 | 14.9 | 5.9 | 19.8 | 21.6 | 20.5 | 5.9 | 14.3 | 10.4 |
| ALB, g/L | 50 | 41 | 40 | 44 | 44 | 33 | 43 | 28 | 50 | 24 | 42 | 44 | 38 |
| CR, μmol/L | 65 | 50 | 107 | 64 | 54 | 79 | 58 | 42 | 76 | 79 | 71 | 66 | 42 |
| BUN, μmol/L | 5.50 | 4.10 | 5.14 | 3.38 | 2.34 | 3.55 | 4.41 | 5.41 | 4.34 | 8.90 | 3.82 | 2.45 | 3.06 |
| PT, s | 13.6 | 13.6 | 13.1 | 12.5 | 11.4 | 12.5 | 11.9 | 17.4 | 12.3 | 11.4 | 12.6 | 12.2 | 13.1 |
| PLT, G/L | 158 | 136 | 128 | 146 | 317 | 201 | 94 | 55 | 122 | 119 | 192 | 167 | 158 |
| HbsAg, (+/-) | + | + | + | + | + | + | + | + | + | + | - | + | + |
| HbeAg, (+/-) | - | - | - | - | - | - | + | + | + | + | - | - | - |
| HBV DNA, copies/ml | ˂1000 | ˂1000 | ˂100 | ˂100 | ˂1000 | ˂100 | ˂100 | 1490 | ˂1000 | 1540 | ˂1000 | ˂1000 | ˂100 |
| BCLC before resection, A/B/C | A | C | A | A | A | A | A | A | A | A | A | A | A |
| Treatment before MASCT | Resection | Resection | Resection | Resection | Resection* | Resection | Resection | Resection | Resection | Resection | Resection | Resection | Resection |

Dates are expressed as the mean ± SD (median) or as the number of patients.

ALT, alanine aminotransferase; AST, aspartate transaminase; TBIL, total bilirubin; ALB, albumin; PLT, platelet count; CR, creatinine; BUN, blood urine nitrogen; AFP, alpha-fetoprotein; CEA, carcino-embryonic antigen; PT, prothrombin time; HBsAg, hepatitis B surface antigen; HbeAg, hepatitis B envelope antigen.

*：Addition TACE treament（transcatheter arterial chemoembolization ）

**Table S3. Demographics and characteristics of HCC patients** **during MASCT treatment**

| **Characteristics** | **MASCT**  **(n=13)** | ***P*^a^** |
| --- | --- | --- |
|  |  |  |
| **AFP, ng/mL** |  | 0.368 |
| W 1 | 11.81 (14.3)^b^ |  |
| W 16 | 9.65 (13.41) |  |
| W 31 | 25.49 (52.49) |  |
| W 52 | 2.87 (2.49) |  |
| **CEA, ng/mL** |  | 0.222 |
| W 1 | 2.79 (2.38) |  |
| W 16 | 2.45 (1.50) |  |
| W 31 | 4.19 (2.53) |  |
| W 52 | 3.00 (2.07) |  |
| **ALT, ng/mL** |  | 0.649 |
| W 1 | 36 (39) |  |
| W 16 | 26 (11) |  |
| W 31 | 35 (13) |  |
| W 52 | 28 (8) |  |
| **AST, ng/mL** |  | 0.684 |
| W 1 | 33 (15) |  |
| W 16 | 29 (10) |  |
| W 31 | 36 (11) |  |
| W 52 | 34 (18) |  |
| **BUN, μmol/L** |  | 0.888 |
| W 1 | 4.34 (1.71) |  |
| W 16 | 4.23 (0.92) |  |
| W 31 | 4.64 (1.11) ^ND^ |  |
| W 52 | 4.28 (1.20) |  |
| **CR, μmol/L** |  | 0.572 |
| W 1 | 66 (18) |  |
| W 16 | 68 (17) |  |
| W 31 | 73 (17) |  |
| W 52 | 75 (17) |  |

| **Characteristics** | **MASCT**  **(n=13)** | ***P*^a^** |
| --- | --- | --- |
| **PLT, G/L** |  | 0.841 |
| W 1 | 153 (63) |  |
| W 16 | 135 (53) |  |
| W 31 | 142 (50) |  |
| W 52 | 137 (45) |  |
| **ALB, g/L** |  | 0.532 |
| W 1 | 40 (8) |  |
| W 16 | 42 (6) |  |
| W 31 | 43 (4) |  |
| W 52 | 43 (4) |  |
| **TBIL, ng/mL** |  | 0.958 |
| W 1 | 11.8 (5.8) |  |
| W 16 | 12.4 (5.9) |  |
| W 31 | 12.1 (4.8) |  |
| W 52 | 11.1 (5.4) |  |

Data are shown as mean (SD)

^a^ One-way ANOVA test was used for statistic

analysis in each group.

^b^ One sample was not detected.

**Table S4. The immune profiles of HCC patients` PBMCs during treatment of MASCT**

| **Immune profiles (%)** | **MASCT** | | | | ***P^a^*** |
| --- | --- | --- | --- | --- | --- |
|  | **W1 (n=13)** | **W16 (n=13)** | **W31 (n=10)** | **W52 (n=7)** |  |
| **CD3** | 71.78±14.72 | 70.95±13.54 | 75.21±10.69 | 57.71±14.64 | 0.072 |
| **CD3^+^CD8^+^** | 25.23±8.86 | 24.25±8.84 | 24.64±10.43 | 20.19 ±7.19 | 0.672 |
| **CD3^+^CD4^+^** | 39.45±10.17 | 42.68±10.22 | 43.45±7.79 | 32.67±7.71 | 0.325 |
| **CD3^+^CD56^+^** | 2.12±1.68 | 2.38±1.92 | 2.05±1.15 | 7.28±8.74 | 0.016^*^ |
| **Treg**^b^ | 2.61±1.19 | 2.20±0.95 | 1.38±0.63 | 0.77±0.45 | 0.000^***^ |
| **CD8^+^NKG2D^+^** | 21.43±11.76 | 21.04±11.43 | 22.36±10.93 | 15.83±10.67 | 0.667 |
| **CD56^+^NKG2D^+^** | 8.41±7.65 | 6.93±5.78 | 6.29±5.14 | 18.57±10.35 | 0.005^***^ |
| **CD8^+^Granzyme B^+^** | 7.78±5.30 | 7.35±4.50 | 6.29±5.42 | 3.93±4.53 | 0.393 |
| **CD8^+^CD107a^+^** | 0.68±0.47 | 0.50±0.49 | 0.91±1.98 | 0.09±0.06 | 0.422 |
| **Effector T cells** ^c^ | 12.68±3.83 | 13.28±5.43 | 13.49±5.33 | 23.53±5.88 | 0.000^***^ |
| **Central memory T cells** ^d^ | 4.83±4.56 | 6.02±5.50 | 6.45±5.57 | 3.17±1.76 | 0.746 |

***^a^*** One-way ANOVA test was used for statistic analysis.

^b^ Tregs were defined as CD4^+^ FoxP3^+^ T cells.

^c^ Effector T cells were defined as CD45RO^-^CCR7^-^CD57^+^ T cells.

^d^ Central memory T cells were defined as CD57^-^ CD27^+^CD45RO^+^ T cells.
